# Oxidative stress and antioxidant pathways in the pathogenesis of periodontitis and peri-implantitis: mechanistic insights and therapeutic potentials

**DOI:** 10.3389/froh.2026.1814823

**Published:** 2026-04-29

**Authors:** Nada Tawfig Hashim, Rasha Babiker, Vivek Padmanabhan, Md Sofiqul Islam, Nallan C. S. K. Chaitanya, Riham Mohammed, Sivan Padma Priya, Ayman Ahmed, Ghiath Mahmoud, Bakri Gobara Gismalla, Muhammed Mustahsen Rahman

**Affiliations:** 1 Department of Periodontics, RAK College of Dental Sciences, RAK Medical & Health Sciences University, Ras-AlKhaimah, United Arab Emirates; 2 Department of Physiology, RAK College of Medical Sciences, RAK Medical & Health Science University, Ras-AlKhaimah, United Arab Emirates; 3 Department of Pediatric and Preventive Dentistry, RAK College of Dental Sciences, RAK Medical & Health Sciences University, Ras-AlKhaimah, United Arab Emirates; 4 Department of Operative Dentistry, RAK College of Dental Sciences, RAK Medical and Health Sciences University, Ras-AlKhaimah, United Arab Emirates; 5 Department of Oral Medicine and Radiology, RAK College of Dental Sciences, RAK Medical & Health Sciences University, Ras-AlKhaimah, United Arab Emirates; 6 Department Oral Surgery, RAK College of Dental Sciences, RAK Medical & Health Sciences University, Ras-AlKhaimah, United Arab Emirates; 7 Oral Pathology Department, RAK College of Dental Sciences, RAK Medical & Health Sciences University, Ras-AlKhaimah, United Arab Emirates; 8 Department of Periodontology and Implantology, Nile University, Khartoum, Sudan; 9 Orthodontics Department, RAK College of Dental Sciences, RAK Medical & Health Sciences University, Ras-AlKhaimah, United Arab Emirates; 10 Department of Oral Rehabilitation, Faculty of Dentistry, University of Khartoum, Khartoum, Sudan

**Keywords:** antioxidants, host modulation, oxidative stress, peri-Implantitis, periodontitis, precision periodontology, reactive oxygen species (ROS), redox biomarkers

## Abstract

**Background:**

Periodontitis and peri-implantitis are chronic, immune-mediated inflammatory diseases characterized by progressive destruction of tooth- and implant-supporting tissues. Although microbial dysbiosis initiates these conditions, accumulating evidence indicates that host-derived oxidative stress plays a central role in amplifying inflammation, impairing tissue repair, and driving irreversible bone loss. Excessive production of reactive oxygen species (ROS) disrupts redox homeostasis, induces molecular damage, and activates redox-sensitive signaling pathways that perpetuate tissue destruction.

**Objective:**

This narrative review synthesizes current mechanistic, clinical, and translational evidence on the role of oxidative stress and antioxidant defense systems in the pathogenesis of periodontitis and peri-implantitis. It further aims to critically evaluate redox-regulated molecular pathways, emerging diagnostic biomarkers, and antioxidant-based therapeutic strategies.

**Methods:**

A structured literature search was conducted using PubMed, Scopus, and Web of Science, focusing on recent experimental, clinical, and translational studies. Articles were selected based on relevance, methodological rigor, and translational applicability, with emphasis on studies addressing oxidative stress mechanisms, biomarker validity, and therapeutic interventions.

**Results:**

Evidence indicates that excessive ROS generation activates key redox-sensitive signaling pathways, including NF-κB, MAPKs, and AP-1, leading to sustained cytokine production, matrix metalloproteinase activation, mitochondrial dysfunction, and enhanced osteoclastogenesis. Concurrent impairment of endogenous antioxidant systems further exacerbates tissue vulnerability. Oxidative stress biomarkers—such as malondialdehyde, 8-hydroxy-2′-deoxyguanosine, and protein carbonyls—demonstrate associations with disease severity and treatment response; however, their clinical utility is limited by methodological heterogeneity and lack of standardization. Antioxidant-based interventions, including systemic supplementation, local delivery systems, nano-formulations, and antioxidant-enriched biomaterials, show promising adjunctive effects, although clinical outcomes remain variable due to differences in bioavailability, dosage, and patient-specific factors.

**Conclusion:**

Oxidative stress represents a central, disease-modifying axis in periodontitis and peri-implantitis. Targeting redox imbalance offers a biologically grounded framework for improving diagnostics, risk stratification, and host-modulatory therapy. However, translation into clinical practice requires standardized biomarker validation and rigorously designed, biomarker-guided clinical trials. Future strategies integrating redox biology with advanced delivery systems and precision medicine approaches may significantly enhance periodontal and peri-implant care.

## 1 Introduction

Periodontitis and peri-implantitis are chronic inflammatory diseases characterized by progressive destruction of the tooth- and implant-supporting tissues, ultimately leading to tooth loss and implant failure (1). Although their microbial etiology is well established, these conditions are now recognized as the result of a complex and dysregulated host–microbe interaction rather than a purely bacterial infection (2). Current understanding emphasizes that microbial dysbiosis triggers a sustained and non-resolving immune-inflammatory response, which becomes the primary driver of connective tissue degradation, alveolar bone resorption, and impaired tissue regeneration (2).

Periodontitis is among the most prevalent non-communicable diseases globally, affecting over one billion individuals, with severe forms ranking among the leading causes of years lived with disability (3, 4). Peri-implantitis, although less extensively quantified, is increasingly recognized as a significant clinical challenge, driven by the widespread use of dental implants, aging populations, and the rising prevalence of systemic comorbidities (5). Together, the substantial clinical and socioeconomic burden of these conditions underscores the need for mechanistically informed therapeutic strategies that extend beyond conventional plaque control and mechanical debridement (1).

While periodontitis and peri-implantitis share several inflammatory and oxidative stress–related mechanisms, they represent biologically distinct tissue environments. In particular, peri-implant tissues lack a periodontal ligament and exhibit differences in vascularization and structural organization, which may influence disease progression and host response (6).

Among the biological processes implicated in periodontal and peri-implant tissue destruction, oxidative stress has emerged as a central and integrative pathogenic mechanism (7). It arises when the production of reactive oxygen species (ROS) exceeds the capacity of endogenous antioxidant defenses, leading to cumulative oxidative damage to lipids, proteins, and nucleic acids (8). While ROS play essential roles in physiological host defense and intracellular signaling, their excessive and sustained generation in inflamed tissues transforms them into potent amplifiers of tissue injury (9).

Within the periodontal microenvironment, neutrophils, macrophages, and activated fibroblasts generate high levels of superoxide anions, hydrogen peroxide, and hydroxyl radicals as part of the innate immune response. These reactive species activate redox-sensitive transcription factors, including nuclear factor kappa B (NF-κB), activator protein-1 (AP-1), and mitogen-activated protein kinases (MAPKs), thereby upregulating pro-inflammatory cytokines, chemokines, and matrix-degrading enzymes (9, 10). Concurrently, ROS directly damage extracellular matrix components, disrupt collagen architecture, and promote osteoclastogenesis, linking inflammation to irreversible tissue breakdown (7, 11). In contrast, the nuclear factor erythroid 2–related factor 2 (Nrf2) pathway functions as a key cytoprotective regulator by inducing the expression of antioxidant and detoxifying enzymes, including superoxide dismutase, catalase, glutathione peroxidase, and heme oxygenase-1 (12). However, growing evidence indicates that this protective system is impaired in periodontitis and peri-implantitis, shifting the balance toward a pro-oxidant and tissue-destructive environment (13–15). Importantly, this redox imbalance extends beyond local tissues, contributing to systemic oxidative stress and supporting established links with conditions such as diabetes mellitus, cardiovascular disease, and neuroinflammatory disorders (11).

Beyond its pathogenic role, oxidative stress represents a promising therapeutic target. Antioxidant-based interventions—including vitamins, endogenous redox modulators, polyphenols, phytochemicals, and nano-formulated delivery systems—are increasingly explored as adjuncts to conventional periodontal and peri-implant therapy (16, 17). These approaches aim not only to suppress inflammation but also to restore redox homeostasis, enhance tissue regeneration, and improve host resilience. In parallel, salivary and gingival crevicular fluid redox biomarkers are emerging as non-invasive tools for early diagnosis, disease stratification, and monitoring of treatment response, supporting the development of precision periodontal care (18).

Against this background, this narrative review aims to synthesize current knowledge on the role of oxidative stress and antioxidant pathways in the pathogenesis of periodontitis and peri-implantitis. It integrates mechanistic insights from molecular biology, immunology, and redox signaling with emerging clinical and translational evidence, with particular emphasis on therapeutic strategies, biomarker development, and future directions toward personalized redox-based interventions.

Although this work is structured as a narrative review, efforts were made to ensure methodological transparency and comprehensive coverage of the literature. A structured search strategy was conducted across PubMed, Scopus, and Web of Science databases, focusing primarily on studies published within the last 10–15 years, while also incorporating seminal earlier contributions of high relevance. Search terms included combinations of “oxidative stress,” “reactive oxygen species,” “periodontitis,” “peri-implantitis,” “antioxidants,” “redox biomarkers,” and “Nrf2 signaling.” Studies were selected based on relevance to mechanistic pathways, clinical correlations, and translational applications, with priority given to clinical studies, systematic reviews, and well-designed experimental models. Articles lacking methodological rigor or direct relevance to periodontal or peri-implant contexts were excluded. This approach was intended to balance comprehensive coverage with critical selection of the most informative and impactful evidence.

## 2 Oxidative stress in periodontal and peri-implant diseases

Oxidative stress refers to a pathological state in which the production of reactive oxygen species (ROS) exceeds the capacity of endogenous antioxidant defense systems, resulting in irreversible molecular damage and dysregulated redox signaling (19). In periodontal and peri-implant tissues, this imbalance functions not merely as a secondary consequence of inflammation but as a key driver of disease initiation, amplification, and chronicity. Sustained ROS accumulation reshapes the inflammatory microenvironment, alters immune cell function, disrupts tissue homeostasis, and accelerates connective tissue degradation and bone resorption (20, 21).

**Figure 1:**
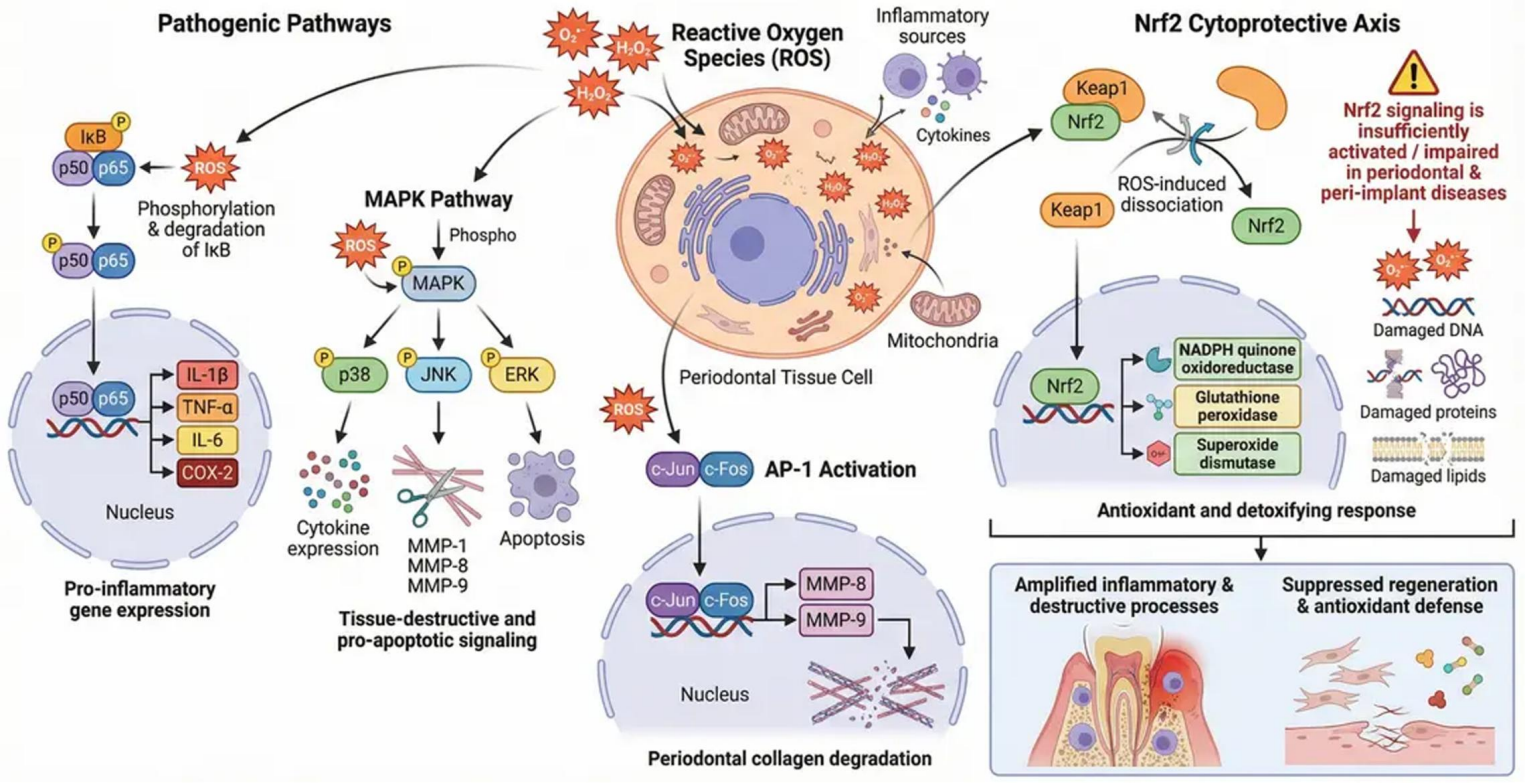
Role of oxidative stress in periodontal and peri-implant tissue homeostasis and disease progression. Under normal conditions, reactive oxygen species (ROS) are produced at physiological levels and balanced by endogenous antioxidant systems, maintaining tissue homeostasis and supporting normal cellular signaling. During microbial challenge, pathogenic biofilms stimulate immune cell activation, leading to excessive ROS production that exceeds antioxidant buffering capacity. This imbalance results in oxidative stress, causing damage to connective tissue, DNA fragmentation, mitochondrial dysfunction, and lipid peroxidation. Persistent oxidative stress promotes inflammation, extracellular matrix degradation, alveolar bone loss, and impaired tissue repair. Sustained ROS generation further amplifies inflammatory pathways, creating a self-perpetuating cycle of oxidative injury and chronic periodontal and peri-implant tissue destruction. This figure was created by the authors using FigureLab.

Under physiological conditions, ROS serve as essential second messengers in cellular signaling, regulating processes such as proliferation, differentiation, apoptosis, and antimicrobial defense. However, in periodontitis and peri-implantitis, persistent microbial challenge induces excessive ROS generation that overwhelms antioxidant buffering capacity. This transition from regulated redox signaling to pathological oxidative stress establishes a self-perpetuating cycle of inflammation, tissue injury, and impaired resolution (7, 9) (Figure 1).

### 2.1 Cellular and molecular sources of ROS in periodontal and peri-implant tissues

The periodontal and peri-implant microenvironments are particularly susceptible to oxidative stress due to continuous exposure to microbial biofilms and mechanical forces. Multiple cellular sources contribute to ROS overproduction, with innate immune cells representing the primary contributors (7, 9). Neutrophils, which are abundant in periodontal lesions, serve as the first line of defense against microbial invasion. Through the respiratory burst, they generate superoxide anions via activation of nicotinamide adenine dinucleotide phosphate (NADPH) oxidase (NOX) complexes (22). Superoxide is subsequently converted into hydrogen peroxide and other highly reactive derivatives, including hypochlorous acid, through myeloperoxidase activity. While this mechanism is essential for microbial clearance, its persistent activation leads to collateral tissue damage, extracellular matrix degradation, and oxidative modification of host proteins (23).

Macrophages and monocytes further amplify ROS generation through the production of nitric oxide (NO) via inducible nitric oxide synthase (iNOS). NO can react with superoxide to form peroxynitrite, a highly cytotoxic reactive nitrogen species that induces lipid peroxidation, protein nitration, and DNA strand breaks, thereby intensifying tissue injury and perpetuating inflammatory cascades (24).

In addition to immune-derived ROS, mitochondrial dysfunction has emerged as a critical intracellular source of oxidative stress in periodontal disease. Chronic inflammatory stimuli disrupt the electron transport chain, resulting in electron leakage and excessive mitochondrial superoxide production. This process not only damages mitochondrial DNA but also promotes mitochondrial-dependent apoptosis, cellular senescence, and inflammasome activation, thereby sustaining inflammatory responses (25).

Beyond immune cells, resident periodontal fibroblasts, osteoblasts, epithelial cells, and endothelial cells actively contribute to redox dysregulation. These cells express NOX isoforms that become hyperactivated in response to bacterial lipopolysaccharides (LPS), mechanical stress, and pro-inflammatory cytokines. As a result, ROS production becomes a multicellular phenomenon, amplifying oxidative damage across periodontal and peri-implant tissues (7, 9).

### 2.2 Microbial dysbiosis and redox perturbation

Periodontal and peri-implant diseases are driven by dysbiotic microbial communities rather than single pathogens. Keystone species such as Porphyromonas gingivalis (*P. gingivalis*), Tannerella forsythia (*T. forsythia*), and Treponema denticola (*T. denticola*) manipulate host immune responses to promote their survival while simultaneously inducing oxidative stress (26). These pathogens activate Toll-like receptors (TLRs) on host cells, triggering downstream signaling pathways that enhance ROS production. Notably, *P. gingivalis* modulates neutrophil function by delaying apoptosis and prolonging ROS release, thereby amplifying inflammatory responses. In addition, bacterial virulence factors can impair antioxidant enzyme activity, weakening host defenses and fostering a pro-oxidant microenvironment that supports microbial persistence (27, 28).

**Figure 2:**
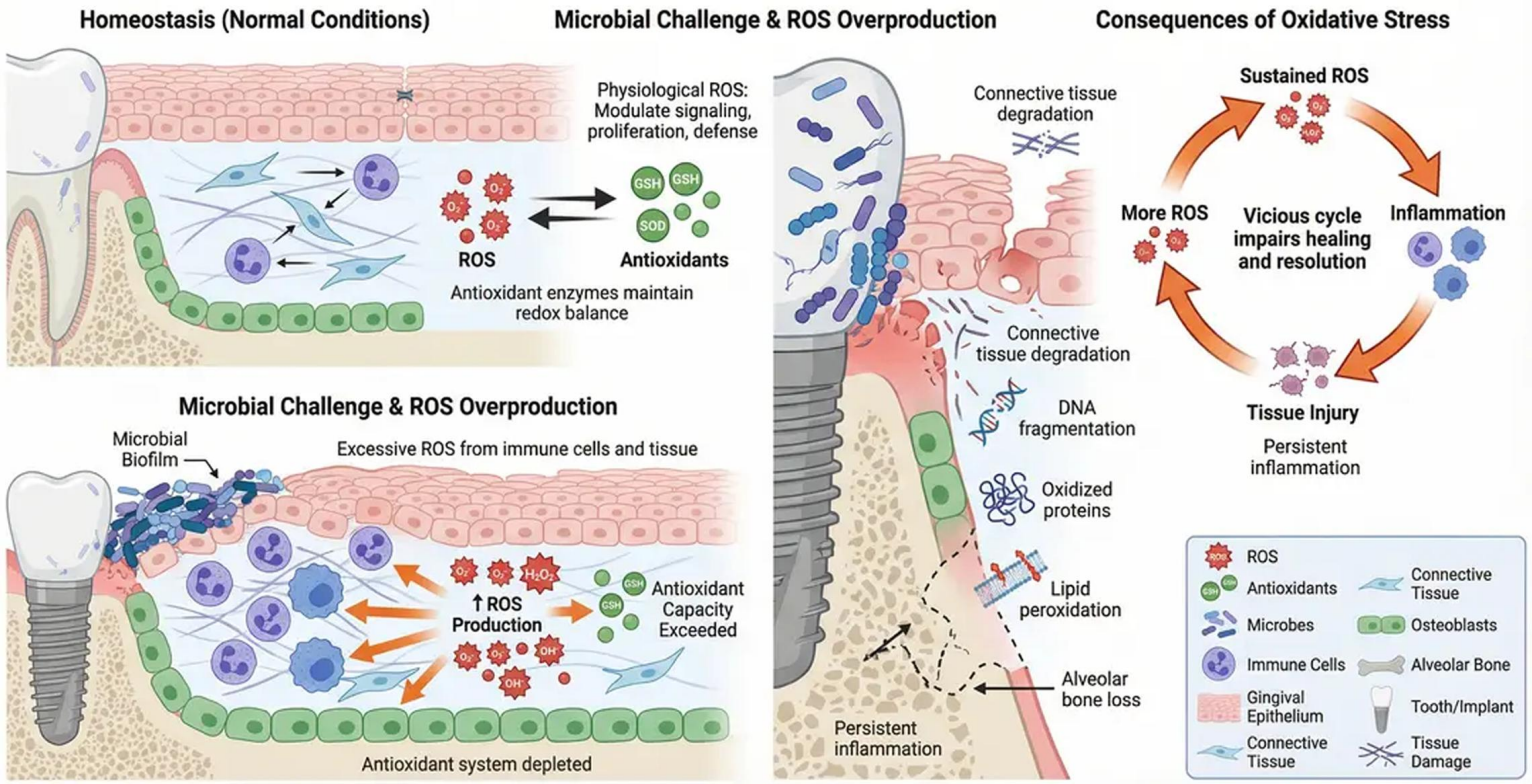
Vicious cycle linking microbial dysbiosis, oxidative stress, and chronic periodontal inflammation. Periodontal dysbiosis, characterized by pathogenic species such as Porphyromonas gingivalis, Tannerella forsythia, and Treponema denticola, activates host immune responses through Toll-like receptors (TLRs) expressed on gingival epithelial cells and immune cells. This activation stimulates intracellular signaling pathways and excessive production of reactive oxygen species (ROS). Neutrophil dysfunction, including delayed apoptosis and prolonged ROS release, further amplifies oxidative stress. Persistent oxidative stress weakens host antioxidant defenses, including superoxide dismutase (SOD), catalase, and glutathione systems, resulting in antioxidant disruption and tissue damage. The resulting pro-oxidant microenvironment promotes microbial persistence and selection of oxidative stress–resistant species, reinforcing microbial dysbiosis and sustaining chronic inflammation. This self-perpetuating cycle contributes to progressive periodontal tissue destruction and impaired host immune regulation. This figure was created by the authors using FigureLab.

Importantly, oxidative stress itself can reshape the microbial ecosystem by selecting for more pathogenic and oxidative stress–resistant species. This bidirectional interaction between dysbiosis and redox imbalance establishes a self-reinforcing cycle in which microbial communities and oxidative stress perpetuate each other, sustaining chronic inflammation (29) (Figure 2).

### 2.3 Redox-Sensitive signaling pathways in periodontal inflammation

ROS exert their pathogenic effects not only through direct molecular damage but also by acting as key regulators of redox-sensitive signaling pathways (30). Among these, nuclear factor kappa B (NF-κB) plays a central role in orchestrating the inflammatory response. ROS activate NF-κB through phosphorylation and degradation of its inhibitory proteins, leading to nuclear translocation and subsequent transcription of pro-inflammatory genes, including IL-1β, TNF-α, IL-6, and cyclooxygenase-2 (31). In parallel, ROS activate mitogen-activated protein kinase (MAPK) pathways—such as p38, JNK, and ERK—which regulate cytokine production, matrix metalloproteinase (MMP) expression, and apoptotic signaling (32). Collectively, these pathways converge to amplify tissue-destructive processes while impairing regenerative responses.

Activator protein-1 (AP-1), another redox-sensitive transcription factor, is activated within oxidative microenvironments and promotes the expression of MMPs, particularly MMP-8 and MMP-9, which are strongly implicated in periodontal collagen degradation (33).

**Figure 3:**
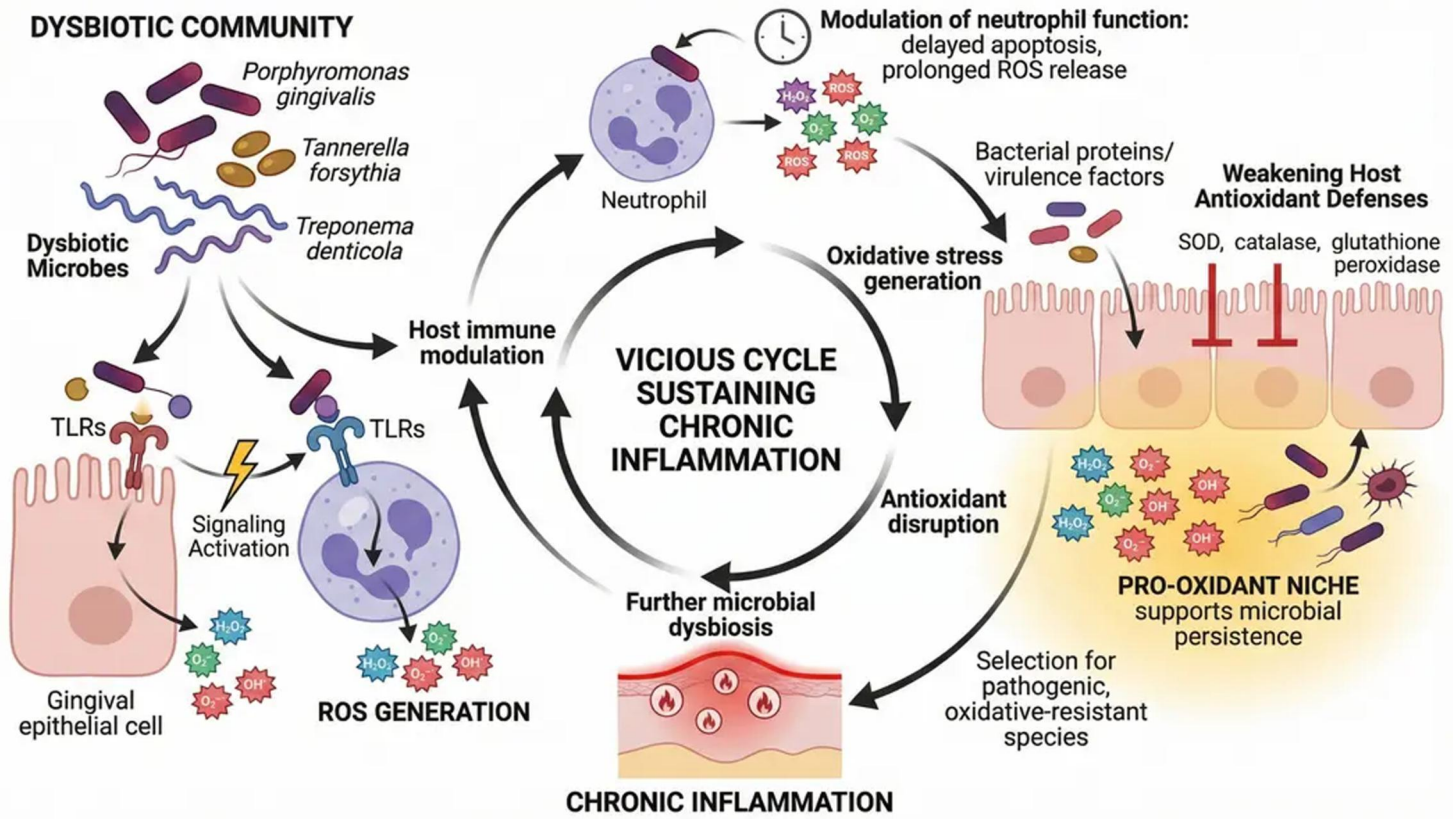
Reactive oxygen species–mediated pathogenic signaling and the Nrf2 cytoprotective axis in periodontal tissue. Excessive production of reactive oxygen species (ROS) activates pathogenic intracellular signaling pathways, including MAPK cascades (p38, JNK, ERK), leading to activation of transcription factors such as NF-κB and AP-1. These pathways promote expression of pro-inflammatory cytokines, matrix metalloproteinases (MMPs), and other mediators responsible for extracellular matrix degradation and periodontal tissue destruction. ROS also induce mitochondrial dysfunction, oxidative damage to proteins and lipids, and amplification of inflammatory responses. The Nrf2 cytoprotective axis functions as a critical antioxidant defense mechanism. Under oxidative stress, Nrf2 dissociates from Keap1 and translocates to the nucleus, where it induces expression of cytoprotective genes, including HO-1, glutathione synthesis enzymes, and detoxifying enzymes. Dysregulation or impairment of Nrf2 signaling results in reduced antioxidant capacity, enhanced oxidative stress, and progressive periodontal tissue damage. This figure was created by the authors using FigureLab.

In contrast, the nuclear factor erythroid 2–related factor 2 (Nrf2) signaling pathway represents the principal cytoprotective axis. Under conditions of oxidative stress, Nrf2 dissociates from its inhibitor Keap1 and translocates to the nucleus, where it induces the transcription of antioxidant and detoxifying genes (34). However, accumulating evidence suggests that Nrf2 signaling is insufficiently activated or functionally impaired in periodontal and peri-implant diseases, thereby permitting sustained oxidative damage and propagation of inflammatory signaling (35) (Figure 3).

### 2.4 Impact of oxidative stress on periodontal and peri-implant tissues

The structural integrity of periodontal and peri-implant tissues is highly vulnerable to oxidative injury. ROS directly oxidize collagen fibers, elastin, and proteoglycans, compromising the biomechanical properties of the gingiva and periodontal ligament. Lipid peroxidation of cellular membranes alters membrane fluidity and disrupts intracellular signaling, while oxidative DNA damage impairs cellular proliferation and repair (36, 37).

Importantly, oxidative stress is a potent driver of osteoclastogenesis. ROS enhance the expression of receptor activator of nuclear factor-kappa B ligand (RANKL) while suppressing osteoprotegerin (OPG), thereby shifting the balance toward bone resorption. In addition, ROS activate inflammasomes and promote pyroptotic cell death, further contributing to bone loss and progressive tissue destruction (38, 39).

In peri-implant tissues, these effects may be amplified due to inherent structural and functional differences, contributing to increased susceptibility to ROS-mediated degeneration and potentially explaining the more aggressive and treatment-resistant nature of peri-implantitis (40).

Building on this, it is important to consider that much of the current understanding of redox dysregulation is derived from periodontitis models. While shared inflammatory mechanisms exist, direct extrapolation to peri-implant tissues should be approached with caution. The distinct tissue architecture and healing dynamics in peri-implant environments may influence both the intensity and resolution of oxidative stress responses, underscoring the need for peri-implant–specific investigations to better define disease mechanisms and optimize therapeutic strategies (6).

### 2.5 Systemic modifiers of local oxidative stress

Systemic conditions such as diabetes mellitus, obesity, smoking, and aging significantly amplify oxidative stress within periodontal tissues. Hyperglycemia promotes mitochondrial ROS overproduction, the formation of advanced glycation end-products (AGEs), and activation of redox-sensitive inflammatory pathways (41, 42). Smoking contributes additional oxidative burden by introducing exogenous free radicals while concurrently suppressing antioxidant enzyme activity (43).

These systemic modifiers not only elevate baseline oxidative stress but also compromise antioxidant defense capacity, thereby lowering the threshold for periodontal breakdown. Collectively, this interplay underscores the need to consider periodontal and peri-implant diseases within the broader context of systemic redox dysregulation rather than as isolated oral conditions.

## 3 Antioxidant defense mechanisms

The biological impact of reactive oxygen species (ROS) is tightly regulated by a complex and multilayered antioxidant defense network that maintains redox homeostasis under physiological conditions (44). In periodontal and peri-implant tissues, this system is essential not only for neutralizing excess free radicals but also for modulating redox-sensitive signaling pathways involved in immune surveillance, tissue repair, and remodeling. Disruption of this regulatory network represents a critical shift in disease pathogenesis, transitioning the local microenvironment from controlled redox signaling to destructive oxidative stress (45).

**Figure 4:**
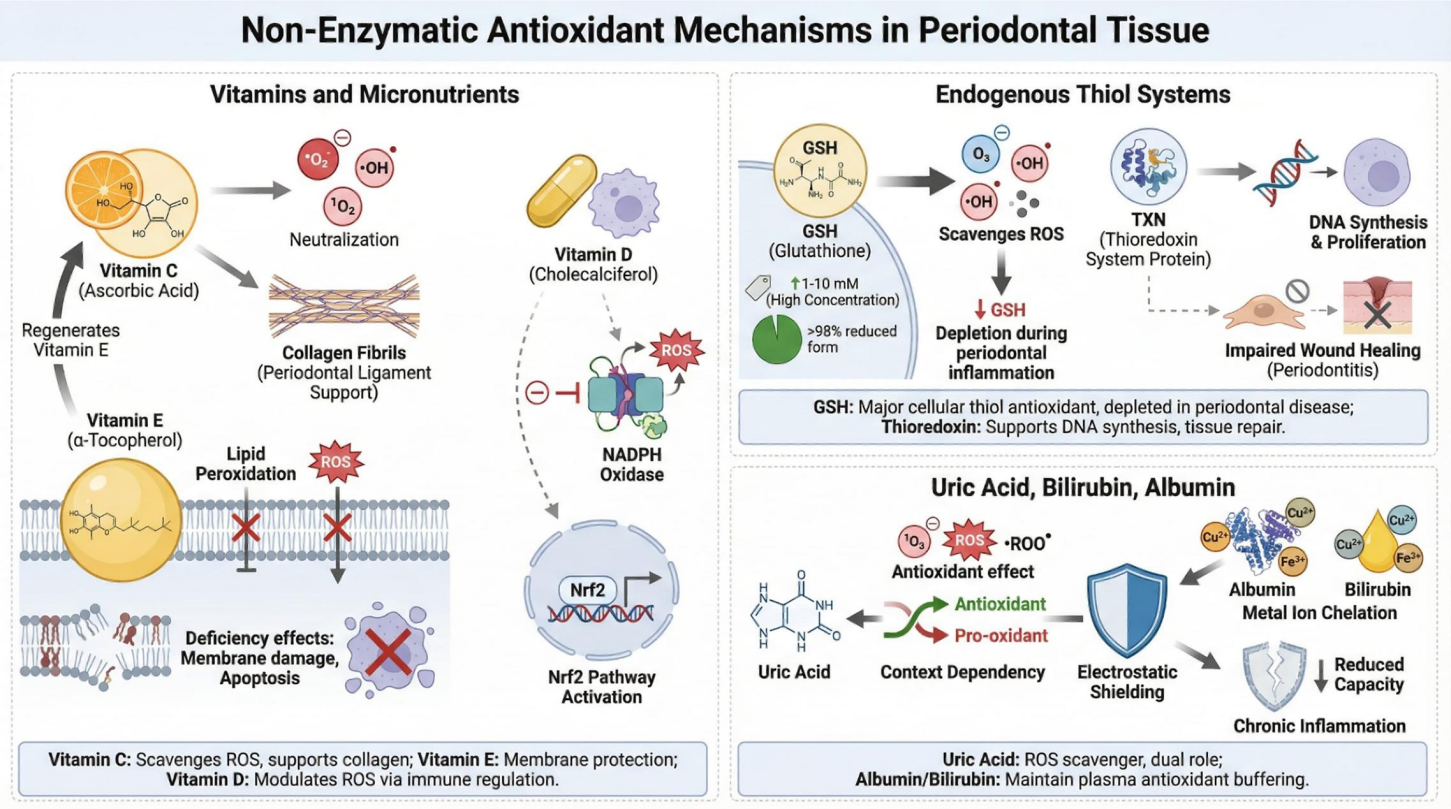
Redox homeostasis and oxidative stress dysregulation in periodontal and peri-implant tissues. Under physiological conditions, reactive oxygen species (ROS) are tightly regulated by endogenous antioxidant systems, including superoxide dismutase (SOD), catalase, glutathione peroxidase (GPx), glutathione, and exogenous antioxidants such as vitamins C and E. These systems maintain redox balance and support immune surveillance, tissue repair, and periodontal homeostasis through redox-sensitive signaling pathways. Loss of antioxidant control leads to excessive ROS accumulation, oxidative stress, and activation of destructive molecular pathways. In periodontitis and peri-implantitis, elevated ROS production promotes inflammatory cell activation, macrophage and neutrophil infiltration, fibroblast dysfunction, and progressive tissue destruction, contributing to periodontal breakdown and peri-implant tissue damage. This figure was created by the authors using FigureLab.

Antioxidant defenses are broadly classified into enzymatic and non-enzymatic systems, which operate in a coordinated and hierarchical manner to ensure efficient detoxification of reactive intermediates while preserving physiological signaling functions (46, 47). In periodontitis and peri-implantitis, however, both components are quantitatively reduced and functionally impaired, rendering tissues highly susceptible to oxidative injury (36) (Figure 4).

### 3.1 Enzymatic antioxidant systems

#### 3.1.1 Superoxide dismutases (SODs)

Superoxide dismutases represent the first line of defense against ROS-mediated damage. These enzymes catalyze the conversion of superoxide anions into hydrogen peroxide and molecular oxygen, thereby preventing the accumulation of highly reactive superoxide radicals. Three major isoforms have been identified: cytosolic Cu/Zn-SOD (SOD1), mitochondrial Mn-SOD (SOD2), and extracellular SOD (SOD3) (48).

In periodontal tissues, SOD activity is critical for protecting gingival fibroblasts, epithelial cells, and osteoblasts from oxidative damage. However, numerous clinical studies have demonstrated reduced SOD levels in gingival crevicular fluid, saliva, and serum of patients with periodontitis. This deficiency leads to unchecked superoxide accumulation, which promotes mitochondrial dysfunction, protein oxidation, and redox-sensitive inflammatory signaling (49, 50).

Particularly relevant is the impairment of mitochondrial SOD2, which exacerbates mitochondrial ROS leakage and contributes to bioenergetic failure, inflammasome activation, and apoptotic signaling. This mitochondrial redox imbalance is increasingly recognized as a driver of chronic inflammation and tissue degeneration in periodontal disease (51).

#### 3.1.2 Catalase (CAT)

Catalase catalyzes the decomposition of hydrogen peroxide into water and oxygen, preventing the formation of highly reactive hydroxyl radicals via the Fenton reaction. This enzyme is abundant in peroxisomes and plays a critical role in buffering oxidative stress under inflammatory conditions (52).

In periodontal and peri-implant lesions, catalase activity is often reduced, either due to oxidative inactivation or transcriptional suppression. This deficiency results in hydrogen peroxide accumulation, which diffuses freely across membranes and oxidizes proteins, lipids, and DNA. Furthermore, hydrogen peroxide functions as a secondary messenger that perpetuates NF-κB activation and cytokine expression, thereby sustaining chronic inflammation (53, 54).

#### 3.1.3 Glutathione peroxidases (GPxs)

The glutathione peroxidase family detoxifies hydrogen peroxide and lipid hydroperoxides using reduced glutathione (GSH) as a cofactor. This system is particularly important for preserving membrane integrity and preventing lipid peroxidation (55).

In periodontal disease, depletion of GSH and reduced GPx activity have been consistently reported. This shift compromises cellular resilience and enhances susceptibility to ferroptosis, a form of regulated cell death driven by lipid peroxidation. Emerging evidence suggests that ferroptotic pathways may contribute to periodontal tissue breakdown, especially under conditions of iron overload and chronic inflammation (56, 57).

#### 3.1.4 Heme oxygenase-1 (HO-1) and phase II detoxification enzymes

Heme oxygenase-1 (HO-1) is an inducible enzyme regulated by the Nrf2 pathway and exerts potent antioxidant, anti-inflammatory, and cytoprotective effects. HO-1 degrades heme into biliverdin, carbon monoxide, and free iron, all of which possess immunomodulatory properties (58).

**Figure 5:**
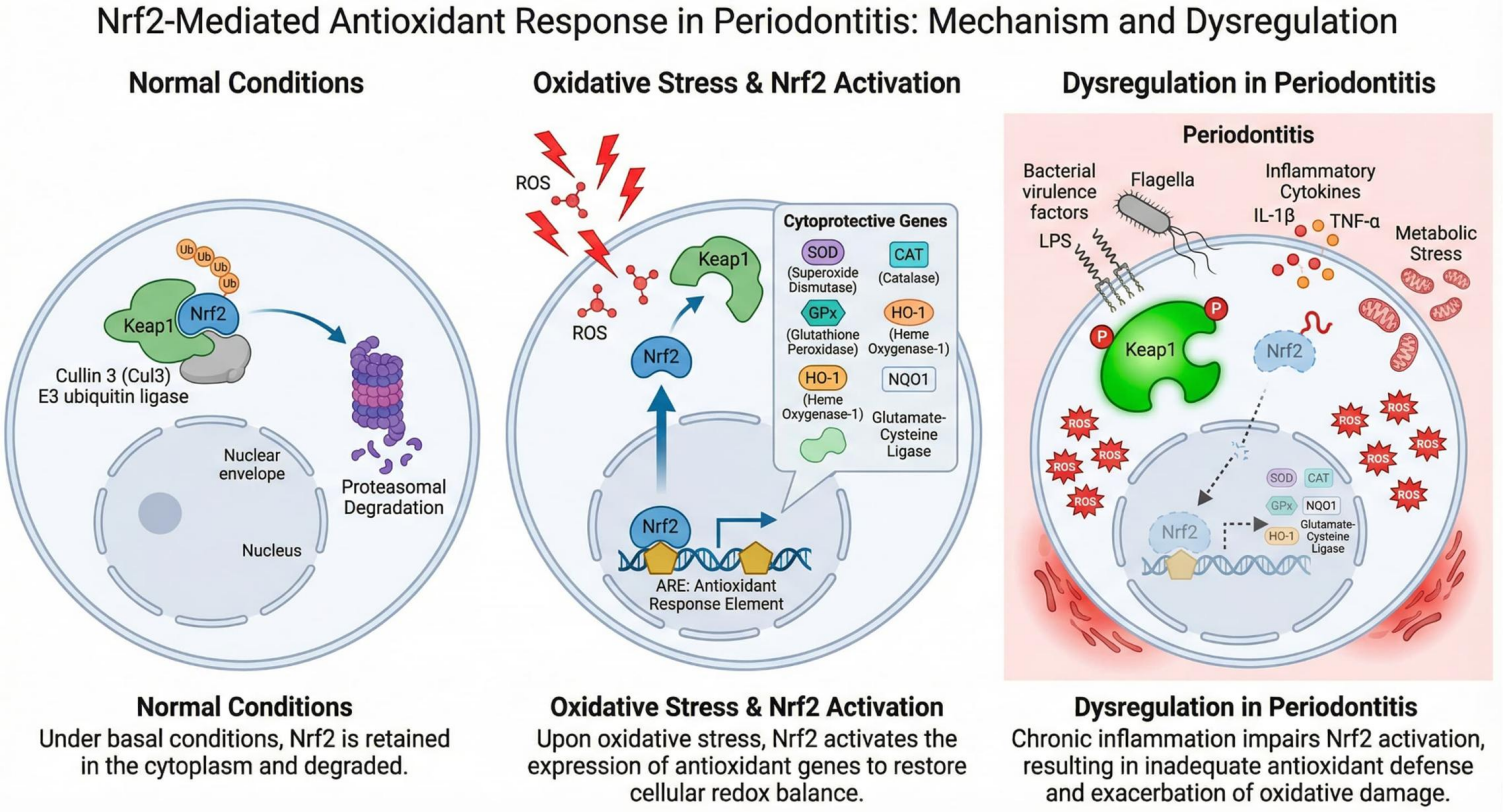
Oxidative stress regulation in healthy periodontal tissue and its dysregulation in periodontitis. In healthy periodontal tissue, balanced antioxidant systems including superoxide dismutase (SOD), catalase (CAT), glutathione peroxidase (GPx), and the Nrf2–HO-1 pathway regulate reactive oxygen species (ROS), preserving mitochondrial function, cellular integrity, and periodontal homeostasis. Gingival fibroblasts, osteoblasts, and immune cells maintain controlled inflammatory responses and tissue repair. In periodontitis, excessive ROS production, mitochondrial dysfunction, lipid peroxidation, and impaired antioxidant defense contribute to ferroptosis, inflammatory cytokine release, extracellular matrix degradation, and progressive periodontal tissue destruction. Dysregulation of Nrf2 signaling and depletion of endogenous antioxidants exacerbate oxidative damage and inflammatory cascades. This figure was created by the authors using FigureLab.

In healthy periodontal tissues, HO-1 is upregulated in response to transient oxidative stress, limiting tissue injury. However, in chronic periodontitis and peri-implantitis, HO-1 induction is often insufficient, delayed, or functionally impaired. This deficiency removes a crucial brake on inflammatory amplification and oxidative damage (7, 9, 58) (Figure 5).

### 3.2 The Nrf2–Keap1 axis: master regulator of redox homeostasis

Nuclear factor erythroid 2–related factor 2 (Nrf2) is the central transcriptional regulator of antioxidant responses. Under basal conditions, Nrf2 is sequestered in the cytoplasm by Kelch-like ECH-associated protein 1 (Keap1), a substrate adaptor protein that facilitates its polyubiquitination by the Cullin 3 (Cul3) E3 ubiquitin ligase, targeting it for proteasomal degradation (59). In response to oxidative stress, Nrf2 dissociates from Keap1 and translocates to the nucleus, where it binds to antioxidant response elements (AREs) and induces the expression of a broad array of cytoprotective genes (60). These include superoxide dismutase (SOD), catalase (CAT), glutathione peroxidase (GPx), heme oxygenase-1 (HO-1), NAD(P)H quinone oxidoreductase 1 (NQO1), and glutamate–cysteine ligase, the rate-limiting enzyme in glutathione synthesis. Through this coordinated transcriptional program, Nrf2 restores redox equilibrium and supports tissue survival (12, 61).

**Figure 6:**
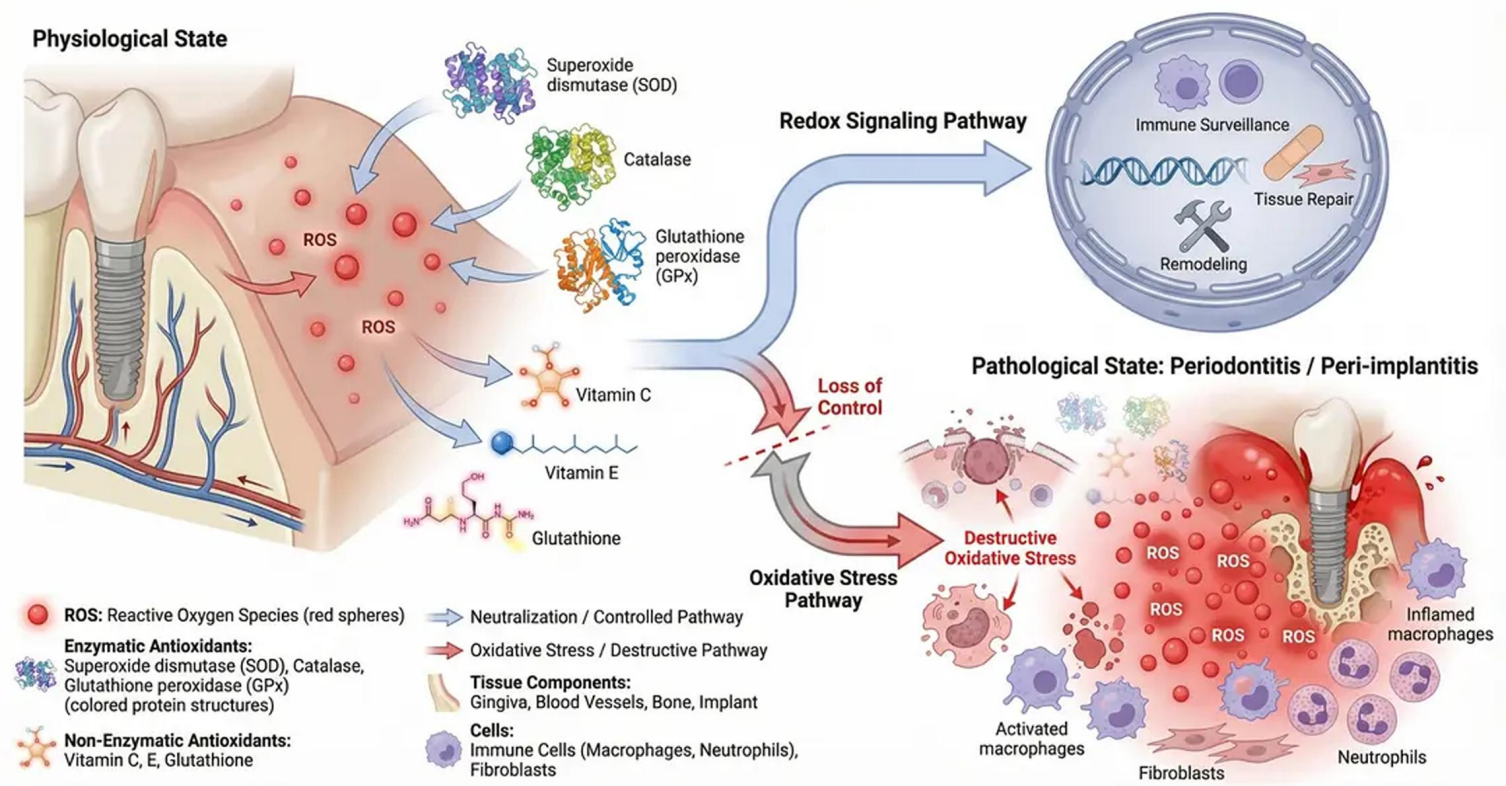
Nrf2–Keap1 signaling pathway under physiological conditions, oxidative stress, and periodontitis. Under basal conditions, Nrf2 is bound by Keap1 in the cytoplasm and targeted for ubiquitin-mediated proteasomal degradation. In response to oxidative stress, reactive oxygen species disrupt the Keap1–Nrf2 interaction, allowing Nrf2 to translocate to the nucleus, where it binds antioxidant response elements (ARE) and induces transcription of cytoprotective genes, including SOD, CAT, HO-1, GPx, and NQO1. In periodontitis, chronic inflammation and persistent oxidative stress impair Nrf2 activation, leading to inadequate antioxidant defense, sustained ROS accumulation, and periodontal tissue damage. This figure was created by the authors using FigureLab.

In periodontitis, however, Nrf2 signaling is frequently dysregulated. Chronic exposure to bacterial virulence factors, inflammatory cytokines, and metabolic stress can impair Nrf2 activation, either through Keap1 hyperactivity or post-translational modifications that destabilize Nrf2. Consequently, the antioxidant response becomes insufficient, allowing oxidative damage to accumulate and sustain inflammatory cascades (62) (Figure 6).

Notably, experimental evidence indicates that pharmacological activation of Nrf2 attenuates alveolar bone loss, reduces inflammatory cytokine expression, and enhances tissue repair, underscoring its potential as a therapeutic target.

### 3.3 Non-enzymatic antioxidant systems

Non-enzymatic antioxidants act as rapid scavengers of free radicals and play a complementary role to enzymatic defenses (47).

#### 3.3.1 Vitamins and micronutrients

Vitamin C (ascorbic acid) is a potent water-soluble antioxidant that neutralizes superoxide, hydroxyl radicals, and singlet oxygen. It also regenerates vitamin E and supports collagen synthesis, making it particularly important for periodontal ligament integrity (63). Clinical studies consistently demonstrate lower plasma and salivary vitamin C levels in patients with periodontitis (64, 65).

Vitamin E (α-tocopherol), a lipid-soluble antioxidant, protects cell membranes from lipid peroxidation. Its deficiency accelerates membrane destabilization, inflammatory signaling, and cell death (66).

Vitamin D, although not a classical antioxidant, exerts indirect antioxidant effects by modulating immune responses, suppressing ROS-generating enzymes, and enhancing Nrf2 signaling (67).

#### 3.3.2 Endogenous thiol systems

Reduced glutathione (GSH) is indeed the most abundant non-protein, small-molecule thiol-based antioxidant present in the cytosol of eukaryotic cells, generally found in millimolar concentrations (1–10 mM). Its high concentration in the reduced form (usually >98% of total glutathione) makes it the primary defense mechanism against oxidative stress and a crucial regulator of cellular homeostasis (68, 69). Periodontal inflammation is associated with marked GSH depletion, reflecting both increased utilization and impaired synthesis (56, 70).

The thioredoxin system also plays a crucial role in redox signaling and DNA synthesis. Its dysregulation contributes to impaired cell proliferation and delayed wound healing in periodontal tissues (71).

#### 3.3.3 Uric acid, bilirubin, and albumin

Uric acid acts as a powerful scavenger of singlet oxygen and peroxyl radicals. Interestingly, elevated systemic uric acid levels may exert paradoxical effects, acting as both an antioxidant and a pro-oxidant depending on the microenvironment (72).

**Figure 7:**
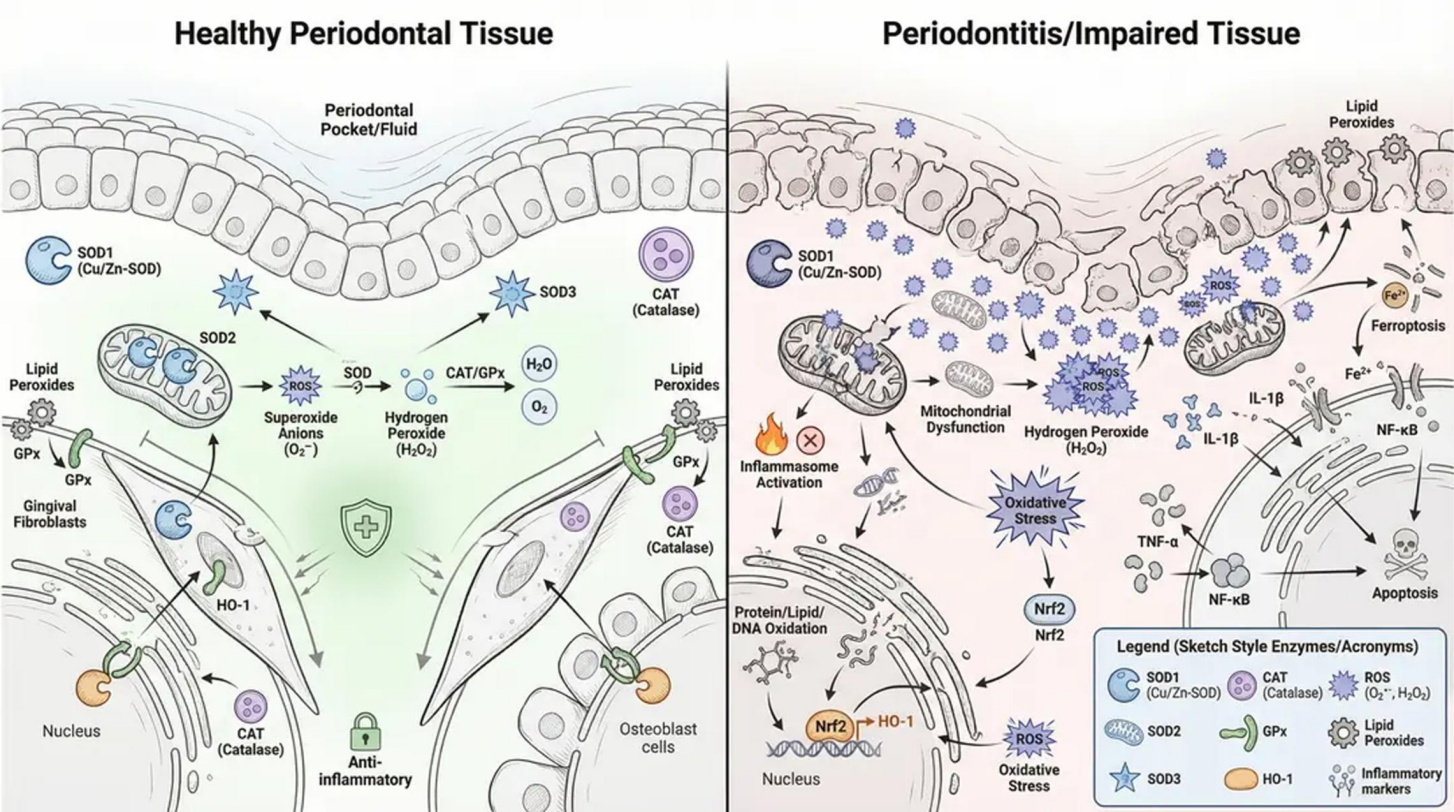
Illustrates the coordinated roles of exogenous micronutrients and endogenous antioxidant systems in maintaining redox homeostasis. (A) Vitamins C, D, and E regulate oxidative stress through membrane stabilization, antioxidant recycling, and activation of protective signaling pathways including Nrf2-mediated transcription. (B) Endogenous thiol systems, including glutathione (GSH/GSSG) and thioredoxin (TXN), detoxify reactive oxygen species and repair oxidatively damaged proteins, supporting periodontal tissue integrity and wound healing. (C) Circulating antioxidants such as uric acid, bilirubin, and albumin contribute to extracellular antioxidant buffering, free radical scavenging, and modulation of inflammatory responses. Together, these systems protect periodontal tissues from oxidative injury and support tissue repair and homeostasis*. This figure was created by the authors using FigureLab.*

Albumin and bilirubin contribute significantly to plasma antioxidant capacity by binding metal ions and neutralizing reactive intermediates. Reduced levels or functional impairment of these molecules further compromises redox buffering in chronic inflammatory states (73) (Figure 7).

Despite encouraging findings from experimental and preclinical studies, the clinical translation of antioxidant-based therapies remains complex and, in some cases, inconsistent. Variability in study outcomes may be attributed to differences in dosage, formulation, route of administration, and treatment duration, as well as inter-individual variability in baseline antioxidant status and genetic factors, including polymorphisms in antioxidant-related pathways and vitamin D receptors (74, 75). In particular, while vitamin D exhibits well-documented immunomodulatory effects, including suppression of pro-inflammatory cytokines and enhancement of antimicrobial peptide expression, clinical studies have reported heterogeneous results regarding its impact on periodontal parameters and redox balance (76, 77). This inconsistency may reflect differences in baseline serum levels, bioavailability, and systemic health status of study populations. Similarly, compounds such as curcumin and melatonin demonstrate potent antioxidant and anti-inflammatory properties *in vitro*; however, their clinical efficacy is often limited by issues related to stability, absorption, and pharmacokinetics (78). Collectively, these considerations underscore the need for rigorously designed randomized controlled trials and biomarker-guided therapeutic approaches to establish the true clinical value of antioxidant interventions in periodontal and peri-implant therapy.

The heterogeneity in clinical outcomes and translational challenges associated with these interventions is further summarized in Table 1.

**Table 1:** Antioxidant-based therapeutic strategies in periodontitis and peri-implantitis: mechanisms, type of evidence, and translational limitations.

Therapeutic agent/strategy	Mechanism of action	Type of evidence	Clinical effects	Strength of evidence	Key limitations	Reference
Vitamin C (Ascorbic acid)	Scavenges ROS; supports collagen synthesis	Clinical + observational	Modest improvement in gingival health	Moderate	Short half-life; dietary variability	(79)
Vitamin E (α-tocopherol)	Prevents lipid peroxidation in membranes	Experimental + limited clinical	Reduces oxidative damage	Low–Moderate	Limited periodontal-specific trials	(80)
Vitamin D	Immunomodulation; enhances antimicrobial peptides; modulates Nrf2 pathway	Clinical + epidemiological	Mixed effects on periodontal parameters	Moderate	Variability in serum levels, VDR polymorphisms, bioavailability	(81)
Curcumin	Inhibits NF-κB; antioxidant and anti-inflammatory	Experimental + small clinical trials	Reduces inflammation and oxidative stress	Moderate	Poor bioavailability; formulation-dependent	(78)
Melatonin	Potent free radical scavenger; regulates mitochondrial function	Experimental + clinical	Improves periodontal healing and reduces inflammation	Moderate	Dose variability; limited long-term studies	(78)
Polyphenols (e.g., resveratrol, green tea catechins)	Antioxidant, anti-inflammatory, modulate signaling pathways	Experimental + clinical	Adjunctive benefits in periodontal therapy	Moderate	Bioavailability and standardization issues	(82)
Coenzyme Q10	Supports mitochondrial function; antioxidant	Clinical (limited)	Improves clinical periodontal parameters	Low–Moderate	Small sample sizes; inconsistent findings	(83)
Nrf2 activators	Upregulate endogenous antioxidant enzymes	Experimental	Reduce oxidative stress and inflammation	Low (preclinical)	Lack of human clinical trials	(84)
Nanoparticle-based antioxidants	Improved delivery and stability	Experimental	Enhanced bioavailability and targeting	Low	Mostly preclinical; safety concerns	(85, 86)
Antioxidant-enriched biomaterials	Promote regeneration and reduce oxidative damage	Experimental	Improved tissue regeneration	Low	Limited clinical translation	(87)

### 3.4 Failure of antioxidant defenses in disease

The breakdown of antioxidant systems in periodontitis and peri-implantitis is multifactorial. Persistent bacterial challenge, chronic cytokine exposure, mitochondrial dysfunction, and metabolic stress progressively exhaust antioxidant reserves. Simultaneously, oxidative modifications of antioxidant enzymes reduce their catalytic efficiency, creating a feed-forward loop of oxidative injury (45, 88). This collapse of redox homeostasis not only accelerates tissue destruction but also interferes with regenerative processes such as fibroblast migration, angiogenesis, and osteoblast differentiation. Consequently, periodontal healing becomes inefficient and incomplete, predisposing to disease recurrence and progression (11, 45).

## 4 Oxidative stress biomarkers in diagnosis and monitoring of periodontitis and peri-implantitis

The recognition of oxidative stress as a central pathogenic mechanism in periodontal and peri-implant diseases has stimulated growing interest in the identification of reliable redox biomarkers for early diagnosis, disease stratification, and monitoring of therapeutic outcomes (89). Unlike traditional clinical parameters, which primarily reflect historical tissue destruction, oxidative stress biomarkers offer a dynamic snapshot of ongoing biological activity, thereby enabling real-time assessment of disease activity and progression (90).

An ideal oxidative stress biomarker should be biologically relevant, sensitive to changes in redox balance, easily accessible through non-invasive sampling, and reproducibly quantifiable. In this regard, saliva, gingival crevicular fluid (GCF), peri-implant sulcular fluid (PISF), and serum represent particularly attractive biological matrices, as they reflect both local and systemic oxidative states (91).

While a growing number of studies report significant associations between oxidative stress biomarkers and periodontal disease severity, the overall strength and consistency of the evidence remain variable. A substantial proportion of available data is derived from cross-sectional designs, which limits the ability to establish temporal or causal relationships between redox imbalance and disease progression. In addition, heterogeneity in sampling protocols, analytical techniques, and biomarker selection contributes to inconsistencies across studies, complicating direct comparisons and meta-analytic synthesis (92–94). Variations in biological matrices, such as saliva, gingival crevicular fluid, and serum—further influence biomarker concentrations and their clinical interpretability. Moreover, systemic conditions, including diabetes, smoking, and dietary antioxidant intake, may act as confounding factors that are not consistently controlled across studies. Therefore, although oxidative stress biomarkers hold considerable promise for diagnostic and prognostic applications, their clinical utility remains contingent upon standardization of methodologies and validation in well-designed longitudinal and interventional studies.

A structured overview of the most relevant oxidative stress biomarkers, including their biological roles, sample sources, and current level of clinical evidence, is provided in Table 2.

**Table 2:** Key oxidative stress biomarkers in periodontitis and peri-implantitis: biological roles, sample sources, and level of clinical evidence.

Biomarker	Biological role	Sample source	Clinical relevance	Level of evidence	Key limitations	Reference
Malondialdehyde (MDA)	End-product of lipid peroxidation; reflects oxidative membrane damage	Saliva, GCF, serum	Correlates with probing depth, CAL, and inflammation; decreases after therapy	Moderate–High (clinical studies available)	Lack of specificity; influenced by systemic oxidative status	(95, 96)
8-Hydroxy-2′-deoxyguanosine (8-OHdG)	Marker of oxidative DNA damage	Saliva, GCF, serum	Associated with disease severity and tissue destruction	Moderate	Variability in assay methods; limited longitudinal data	(97)
Protein carbonyls	Indicator of protein oxidation	Saliva, serum	Reflects cumulative oxidative protein damage	Low–Moderate	Limited periodontal-specific studies	(98)
Total Antioxidant Capacity (TAC)	Represents overall antioxidant defense status	Saliva, serum	Reduced in periodontitis; increases after treatment	Moderate	Non-specific; influenced by diet and systemic factors	(99, 100)
Superoxide dismutase (SOD)	Enzymatic antioxidant converting superoxide radicals	GCF, saliva, serum	Decreased levels associated with oxidative imbalance	Moderate	Enzyme activity varies with local and systemic conditions	(49)
Catalase (CAT)	Detoxifies hydrogen peroxide	GCF, saliva	Reduced activity linked to increased oxidative stress	Low–Moderate	Limited standardized clinical data	(101)
Glutathione (GSH)	Major intracellular antioxidant and redox regulator	GCF, saliva, serum	Depletion associated with disease progression	Moderate	Sensitive to sample handling and storage	(102)
Myeloperoxidase (MPO)	Neutrophil-derived enzyme producing reactive oxidants	GCF, saliva	Marker of inflammatory oxidative activity	High (well-established inflammatory marker)	Reflects inflammation more than specific oxidative damage	(103)
F2-Isoprostanes	Stable products of lipid peroxidation	Serum, saliva	Reliable indicator of *in vivo* oxidative stress	Low–Moderate (limited periodontal data)	Underexplored in periodontal context	(104, 105)
Nitric oxide (NO)/Peroxynitrite	Reactive nitrogen species contributing to oxidative damage	GCF, saliva	Associated with inflammatory burden and tissue injury	Moderate	Dual role (protective vs. damaging) complicates interpretation	(106, 107)

### 4.1 Lipid peroxidation biomarkers

Lipid peroxidation is one of the earliest and most destructive consequences of excessive ROS production. Polyunsaturated fatty acids within cellular membranes are highly susceptible to oxidative attack, generating a variety of secondary reactive aldehydes that further propagate oxidative damage (108).

#### 4.1.1 Malondialdehyde (MDA)

Malondialdehyde (MDA) is the most extensively studied lipid peroxidation product in periodontal research. Elevated MDA levels have been consistently reported in saliva, GCF, and serum of patients with periodontitis and peri-implantitis, correlating positively with probing depth, clinical attachment loss, and bleeding on probing (50, 109).

Mechanistically, MDA forms adducts with proteins and DNA, altering their structure and function. These adducts can act as neoantigens, further stimulating immune responses and perpetuating inflammation (110). Importantly, MDA levels decline following successful periodontal therapy, suggesting its potential utility as a treatment response biomarker (111).

#### 4.1.2 Isoprostanes

F2-isoprostanes are stable end-products of arachidonic acid oxidation and are considered among the most reliable markers of *in vivo* oxidative stress (112). Their formation is independent of enzymatic pathways, making them particularly reflective of free radical-mediated lipid peroxidation. Although less extensively explored in periodontology compared to MDA, emerging studies suggest that isoprostanes may offer superior specificity and stability, positioning them as promising candidates for future diagnostic platforms (113).

### 4.2 DNA oxidation biomarkers

DNA damage represents a critical consequence of chronic oxidative stress, contributing to genomic instability, impaired cellular function, and dysregulated immune responses (114).

#### 4.2.1 8-hydroxy-2′-deoxyguanosine (8-OHdG)

8-Hydroxy-2′-deoxyguanosine (8-OHdG) is widely recognized as the most frequently detected and studied marker of oxidative DNA damage, resulting from the attack of reactive oxygen species, specifically hydroxyl radicals on the C8 position of guanine residues. As a stable end-product of this oxidative modification, 8-OHdG is excreted in biological fluids, making it a crucial, non-invasive indicator for monitoring systemic and localized oxidative stress (97, 115). Multiple clinical studies have demonstrated significantly elevated 8-OHdG levels in patients with periodontitis and peri-implantitis. Importantly, its concentration correlates with disease severity, inflammatory burden, and alveolar bone loss (116, 117). Beyond being a passive indicator of damage, 8-OHdG may actively contribute to pathogenesis by activating pattern recognition receptors and inflammasomes. The detection of 8-OHdG in saliva offers a non-invasive and patient-friendly approach, opening avenues for chairside diagnostics and longitudinal disease monitoring.

### 4.3 Protein oxidation biomarkers

Proteins are highly susceptible to oxidative modifications, which alter their structure, enzymatic activity, and immunogenicity (118).

#### 4.3.1 Protein carbonyls

Protein carbonylation represents an irreversible oxidative modification and serves as a robust marker of cumulative oxidative injury. Increased protein carbonyl levels have been reported in the gingival tissues and saliva of periodontitis patients, reflecting chronic exposure to ROS.

Carbonylated proteins exhibit impaired functionality and resistance to proteolytic degradation, leading to their accumulation and further cellular stress (119, 120).

#### 4.3.2 Advanced oxidation protein products (AOPPs)

AOPPs are generated by chlorinated oxidants, primarily hypochlorous acid, which is abundantly produced by activated neutrophils. Elevated AOPP levels have been associated with severe periodontitis and systemic inflammatory burden (121, 122).

These molecules act as pro-inflammatory mediators by activating NF-κB signaling and promoting cytokine release, thereby linking oxidative damage to immune dysregulation (123).

### 4.4 Total antioxidant capacity (TAC) and redox balance indices

Total Antioxidant Capacity (TAC) is a crucial biomarker used to evaluate the overall ability of biological fluids, such as plasma, serum, and saliva, to neutralize free radicals (ROS). Rather than measuring individual antioxidant components—which can be labor-intensive, costly, and fail to account for synergistic effects—TAC provides a single, integrated measure of all antioxidants present, including their collective, synergistic, and antagonistic interactions (124, 125). Clinical studies consistently show reduced TAC in saliva, GCF, and serum of individuals with periodontitis and peri-implantitis. This reduction reflects both the depletion of antioxidant reserves and the overwhelming burden of oxidative stress (126, 127).

Several assays, including ferric reducing antioxidant power (FRAP), oxygen radical absorbance capacity (ORAC), and trolox equivalent antioxidant capacity (TEAC), have been used to quantify TAC. Among these, FRAP has gained popularity due to its simplicity and reproducibility (128, 129).

The ratio between oxidative damage markers and TAC may offer a more biologically meaningful index of disease activity than either parameter alone, representing the net redox status.

### 4.5 Clinical correlations and prognostic potential

A growing body of evidence suggests that oxidative stress biomarkers are not merely passive indicators but active participants in disease progression. Elevated oxidative damage markers are associated with rapid attachment loss, increased bone resorption, and poor response to conventional therapy. Longitudinal studies demonstrate that individuals with persistently high oxidative burden are more likely to experience disease recurrence and implant failure (53, 130). This highlights the potential of redox biomarkers in identifying high-risk patients who may benefit from intensified or personalized therapeutic strategies.

### 4.6 Toward chairside diagnostics and precision periodontology

The integration of oxidative stress biomarkers into clinical practice holds transformative potential. Advances in biosensor technology, microfluidics, and nanodiagnostics have paved the way for the development of rapid, low-cost, and highly sensitive chairside assays capable of detecting salivary and GCF-based redox markers (131, 132). Such platforms could enable:

- Early disease detection before irreversible tissue destruction
- Real-time monitoring of treatment response
- Risk stratification and personalized therapy
- Improved patient compliance and motivation

Ultimately, redox biomarker-guided approaches could redefine periodontal and peri-implant care, shifting from reactive treatment to proactive disease management.

## 5 Therapeutic modulation of oxidative stress in periodontitis and peri-implantitis

The recognition of oxidative stress as a central pathogenic driver of periodontal and peri-implant tissue destruction has fundamentally reshaped therapeutic paradigms. Traditional mechanical debridement and antimicrobial strategies, while essential, primarily address the microbial component of disease and do not directly target the dysregulated host redox state (7, 133). This limitation may partly explain the persistence of inflammation, incomplete tissue regeneration, and frequent disease recurrence observed in susceptible individuals.

Therapeutic modulation of oxidative stress represents a rational and biologically grounded adjunctive strategy aimed at restoring redox homeostasis, dampening destructive inflammatory cascades, and promoting tissue repair (134). This approach aligns with the broader concept of host modulation therapy, which seeks to rebalance dysregulated immune responses rather than merely suppressing microbial load.

### 5.1 Systemic antioxidant supplementation

Systemic antioxidants have been extensively explored as adjuncts to conventional periodontal therapy. These agents exert pleiotropic effects, including direct free radical scavenging, enhancement of endogenous antioxidant enzyme activity, and suppression of redox-sensitive inflammatory pathways (135).

#### 5.1.1 Vitamins and micronutrients

Vitamin C plays a central role in collagen synthesis, immune regulation, and ROS neutralization. Clinical studies have consistently demonstrated that vitamin C supplementation improves gingival bleeding indices, reduces oxidative stress markers, and enhances periodontal wound healing. Its deficiency is strongly associated with periodontal breakdown, impaired fibroblast function, and delayed tissue repair (136, 137).

Vitamin E, a lipid-soluble antioxidant, protects cellular membranes from lipid peroxidation and stabilizes mitochondrial integrity. Supplementation has been shown to reduce oxidative biomarkers and attenuate inflammatory responses in periodontal tissues, although clinical outcomes remain heterogeneous due to variations in dosage, duration, and baseline antioxidant status (138).

Vitamin D, although not a classical antioxidant, modulates redox balance indirectly through immunomodulatory and anti-inflammatory mechanisms. It suppresses ROS-generating enzymes, enhances Nrf2 signaling, and promotes osteoblastic differentiation, making it particularly relevant in peri-implant bone homeostasis (139).

#### 5.1.2 Polyphenols and natural compounds

Resveratrol, quercetin, catechins, and other polyphenolic compounds exert potent antioxidant and anti-inflammatory effects by scavenging free radicals, inhibiting NF-κB activation, and enhancing Nrf2-mediated cytoprotective pathways. Preclinical and early clinical studies indicate that these compounds reduce alveolar bone loss, suppress inflammatory cytokine production, and improve periodontal parameters when used as adjuncts (140, 141). However, systemic antioxidant supplementation faces challenges, including limited bioavailability, rapid metabolism, and inter-individual variability in response. These limitations have catalyzed interest in localized and advanced delivery strategies (140).

### 5.2 Local antioxidant delivery systems

Local antioxidant therapy offers several advantages over systemic administration, including higher local concentrations, reduced systemic side effects, and site-specific targeting.

Topical formulations, such as antioxidant-containing gels, mouthrinses, and biodegradable chips, have demonstrated promising results in reducing oxidative stress biomarkers and improving clinical outcomes. Coenzyme Q10, for example, has been extensively studied due to its dual role in mitochondrial bioenergetics and antioxidant defense. Local CoQ10 application has been associated with reduced probing depths, improved attachment levels, and decreased inflammatory burden (142, 143).

Curcumin-based gels represent another compelling example. Curcumin exerts strong antioxidant, anti-inflammatory, and antimicrobial effects. It inhibits ROS production, downregulates NF-κB signaling, and promotes fibroblast migration and angiogenesis. Clinical trials indicate that curcumin formulations can enhance the outcomes of scaling and root planing (144, 145).

In peri-implantitis, local delivery becomes even more critical due to the anatomical complexity of peri-implant pockets and the reduced vascularity of peri-implant tissues. Controlled-release systems capable of sustained antioxidant delivery may help counteract the aggressive oxidative microenvironment characteristic of peri-implant lesions (146).

### 5.3 Nanotechnology-based antioxidant delivery

Nanotechnology has emerged as a transformative platform for antioxidant-based periodontal therapy. Nanocarriers can encapsulate antioxidants, protect them from degradation, and facilitate targeted, sustained release (147).

Lipid-based nanoparticles, polymeric nanospheres, and mesoporous silica nanoparticles have been engineered to deliver polyphenols, vitamins, and enzymatic antioxidants directly to inflamed periodontal sites. These systems enhance bioavailability, prolong retention time, and improve cellular uptake (148). Importantly, some nanomaterials possess intrinsic antioxidant properties. Cerium oxide nanoparticles, for instance, exhibit redox cycling between Ce³⁺ and Ce⁴⁺ states, enabling continuous scavenging of superoxide and hydrogen peroxide (149). Preclinical studies demonstrate that these nanozymes reduce inflammatory cytokine expression, suppress osteoclastogenesis, and preserve alveolar bone (150, 151). Moreover, smart nanocarriers responsive to pH, ROS concentration, or enzymatic activity are being developed to enable on-demand drug release, aligning with the principles of precision medicine (152).

### 5.4 Antioxidant-enriched biomaterials and regenerative scaffolds

Periodontal and peri-implant regeneration requires not only suppression of inflammation but also restoration of the biological microenvironment conducive to tissue repair. Oxidative stress severely impairs the regenerative capacity of periodontal ligament stem cells, osteoblasts, and endothelial cells (153). To address this challenge, biomaterials enriched with antioxidant compounds have been developed. Hydrogels, collagen matrices, and bioactive scaffolds incorporating polyphenols, melatonin, or Nrf2 activators can provide localized redox buffering while supporting cell adhesion, proliferation, and differentiation (154).

Melatonin deserves particular attention due to its strong antioxidant capacity, immunomodulatory effects, and osteogenic potential. Locally delivered melatonin has been shown to reduce oxidative stress, enhance bone formation, and improve implant osseointegration in experimental models.

These multifunctional scaffolds represent a new generation of periodontal biomaterials that actively modulate the inflammatory microenvironment rather than serving as passive structural supports (155, 156).

### 5.5 Phytochemicals as host-modulatory agents

Plant-derived compounds constitute a rich source of multifunctional antioxidants with host-modulatory properties. Curcumin, green tea catechins, gingerols, and anthocyanins exert synergistic effects by simultaneously targeting oxidative stress, inflammation, and microbial virulence. These compounds modulate multiple signaling pathways, including NF-κB, MAPK, and Nrf2, thereby suppressing pro-inflammatory mediators while enhancing antioxidant enzyme expression (157–159). This multi-targeted action is particularly advantageous in complex diseases such as periodontitis, where multiple pathogenic mechanisms converge. However, the clinical translation of phytochemicals is limited by poor solubility, rapid metabolism, and low bioavailability. Advanced formulation strategies, including nanoencapsulation and mucoadhesive systems, are actively being explored to overcome these barriers (159).

### 5.6 Integration with conventional therapy

Antioxidant-based interventions should not be viewed as standalone treatments but as biologically informed adjuncts to mechanical debridement, antimicrobial therapy, and regenerative procedures.

By reducing oxidative burden, antioxidants may:

- Enhance wound healing
- Improve stem cell survival
- Increase responsiveness to regenerative cues
- Reduce postoperative inflammation
- Decrease recurrence rates

This integrative approach aligns with the modern concept of periodontal medicine, which emphasizes systemic health, personalized therapy, and long-term disease control (160, 161).

## 6 Oxidative stress and systemic links

Periodontitis and peri-implantitis are increasingly recognized not as isolated oral diseases but as manifestations of systemic inflammatory and redox dysregulation. Oxidative stress represents a common biological denominator linking periodontal inflammation to a wide range of systemic disorders, including diabetes mellitus, cardiovascular disease, neurodegeneration, metabolic syndrome, and aging. This bidirectional relationship is driven by shared molecular pathways involving ROS overproduction, mitochondrial dysfunction, immune dysregulation, and impaired antioxidant defenses (11, 162). From a pathophysiological perspective, periodontal tissues are uniquely positioned at the interface between the external environment and systemic circulation. Chronic periodontal inflammation facilitates the translocation of inflammatory mediators, oxidized biomolecules, and microbial products into the bloodstream, thereby contributing to systemic oxidative burden. Conversely, systemic oxidative stress primes periodontal tissues toward exaggerated inflammatory responses, accelerating tissue destruction and impairing healing (11).

### 6.1 Diabetes mellitus and redox dysregulation

The relationship between diabetes and periodontitis is one of the most extensively studied examples of bidirectional disease interaction. Hyperglycemia promotes excessive mitochondrial ROS production, activation of NADPH oxidases, and formation of advanced glycation end-products (AGEs). These AGEs interact with their receptors (RAGE) on immune and endothelial cells, triggering sustained NF-κB activation and amplifying inflammatory signaling (163).

In periodontal tissues, this pro-oxidant environment enhances neutrophil hyper-reactivity, suppresses fibroblast function, and impairs osteoblast differentiation. ROS-driven activation of RANKL further accelerates alveolar bone resorption, explaining the increased severity and treatment resistance of periodontitis in diabetic individuals. Importantly, periodontal inflammation itself exacerbates systemic oxidative stress by releasing cytokines such as TNF-α and IL-6, which impair insulin signaling and promote further ROS production. This establishes a vicious cycle in which redox imbalance sustains both metabolic dysregulation and periodontal breakdown (9, 35).

Peri-implant tissues appear particularly vulnerable in diabetic patients due to impaired angiogenesis, reduced antioxidant capacity, and delayed wound healing. These factors may contribute to the increased risk of peri-implantitis and implant failure observed in this population (164).

### 6.2 Cardiovascular disease and endothelial dysfunction

Oxidative stress plays a central role in the pathogenesis of atherosclerosis, hypertension, and heart failure. Periodontal inflammation contributes to this process by increasing systemic levels of ROS, oxidized low-density lipoproteins (oxLDL), and pro-inflammatory mediators. Periodontal pathogens and their virulence factors stimulate endothelial cells to produce ROS, reducing nitric oxide bioavailability and impairing vasodilation. This endothelial dysfunction represents an early and reversible stage of cardiovascular disease (35, 165).

Moreover, oxidative modification of circulating lipoproteins enhances their uptake by macrophages, promoting foam cell formation and plaque development. Periodontitis has been associated with elevated systemic markers of lipid peroxidation and reduced antioxidant capacity, providing a mechanistic basis for its association with cardiovascular morbidity (11, 166).

Therapeutically, periodontal treatment has been shown to reduce systemic oxidative stress markers and improve endothelial function, highlighting the systemic benefits of local redox modulation (11).

### 6.3 Neuroinflammation and cognitive decline

Emerging evidence suggests that chronic peripheral inflammation and oxidative stress contribute to neurodegenerative processes. Periodontitis has been associated with increased risk of cognitive impairment, Alzheimer's disease, and Parkinson's disease (167, 168).

Systemic ROS and inflammatory mediators derived from periodontal tissues can disrupt the blood–brain barrier, activate microglia, and promote neuroinflammation. Oxidative damage to neuronal membranes, mitochondrial DNA, and synaptic proteins accelerates neurodegeneration.

Notably, periodontal pathogens such as P. gingivalis have been detected in brain tissues, where they may further induce local ROS production and immune activation. This interplay between microbial challenge and redox imbalance underscores the systemic implications of oral oxidative stress (169, 170).

### 6.4 Aging, immunosenescence, and redox imbalance

Aging is characterized by a progressive decline in antioxidant capacity, accumulation of mitochondrial mutations, and increased basal ROS production—a phenomenon often described as “inflammaging.” This state predisposes older individuals to exaggerated inflammatory responses and impaired tissue regeneration. In periodontal tissues, aging-related redox dysregulation reduces the resilience of fibroblasts, stem cells, and endothelial cells, impairing wound healing and osseointegration (171, 172). This may explain the higher prevalence and severity of periodontitis and peri-implantitis in elderly populations. Furthermore, senescent cells secrete a pro-inflammatory and pro-oxidant secretome known as the senescence-associated secretory phenotype (SASP), which further amplifies local and systemic oxidative stress (173).

### 6.5 Obesity, metabolic syndrome, and redox burden

Adipose tissue is an active endocrine organ that produces pro-inflammatory cytokines and ROS. Obesity is associated with elevated systemic oxidative stress, mitochondrial dysfunction, and impaired antioxidant defenses (174).

In periodontal tissues, these systemic alterations prime immune cells toward a hyper-inflammatory phenotype, enhancing ROS production and tissue destruction. Clinical studies demonstrate that obese individuals exhibit higher levels of oxidative damage biomarkers and poorer periodontal outcomes.

These findings emphasize the need for integrative therapeutic strategies that address both local and systemic redox imbalance (175–177).

### 6.6 Implications for peri-implant health

Compared to natural teeth, peri-implant tissues exhibit reduced vascularity, altered immune surveillance, and limited regenerative capacity. These features make them particularly susceptible to oxidative injury. Systemic redox dysregulation can compromise osseointegration, increase susceptibility to biofilm-induced inflammation, and impair peri-implant wound healing (154, 155). This underscores the importance of systemic risk assessment and antioxidant support in implant therapy.

Future implant protocols may benefit from incorporating redox profiling and antioxidant-based interventions to optimize long-term outcomes.

## 7 Future directions

The expanding body of evidence linking oxidative stress to the pathogenesis of periodontitis and peri-implantitis provides a compelling rationale for re-examining current diagnostic and therapeutic frameworks. While oxidative stress is increasingly recognized as a key disease-modifying factor, its integration into clinical practice remains largely conceptual and investigational. Future strategies should therefore aim to bridge mechanistic insights with clinically validated applications, with careful consideration of translational limitations.

### 7.1 Precision redox medicine in periodontal care

Precision medicine aims to tailor therapeutic interventions based on individual biological profiles rather than relying on uniform treatment protocols. In periodontology, this approach has traditionally focused on genetic susceptibility, microbial signatures, and systemic risk factors. Redox profiling may represent an additional and dynamic layer of personalization; however, its clinical applicability remains preliminary and requires further validation.

Quantification of oxidative damage markers (e.g., MDA, 8-OHdG, AOPPs) and antioxidant capacity indices has been proposed as a means to classify patients into distinct redox phenotypes. While conceptually promising, such stratification currently lacks standardized thresholds, reproducibility across populations, and prospective clinical validation. Consequently, its role in guiding therapeutic decision-making should be considered exploratory rather than established.

### 7.2 Integration of artificial intelligence and redox biomarker analytics

Artificial intelligence (AI) and machine learning have the potential to enhance periodontal diagnostics by integrating complex datasets derived from clinical parameters, imaging, microbiome profiles, and redox biomarkers. However, the application of AI in this context remains in early developmental stages.

Although predictive models may theoretically identify patterns associated with oxidative stress and disease progression, current evidence is limited by small datasets, lack of external validation, and variability in biomarker measurement. The concept of real-time redox monitoring through AI-assisted platforms and biosensors is promising but should be regarded as an emerging research direction rather than an imminent clinical tool.

### 7.3 Smart and stimuli-responsive drug delivery systems

Next-generation antioxidant delivery systems, including stimuli-responsive platforms, offer innovative approaches to improving therapeutic specificity and bioavailability. These systems are being engineered to release therapeutic agents in response to environmental triggers such as elevated ROS levels, acidic pH, or inflammatory mediators.

While preclinical findings are encouraging, the majority of these technologies remain experimental, with limited human data regarding safety, long-term stability, and clinical efficacy. Their translation into routine periodontal and peri-implant therapy will require rigorous clinical evaluation and regulatory validation.

### 7.4 Multi-target host modulation strategies

Given the multifactorial nature of periodontitis and peri-implantitis, multi-target therapeutic strategies combining antioxidants with anti-inflammatory and regenerative agents represent a rational approach. However, evidence supporting synergistic clinical benefits remains inconsistent, and optimal combinations, dosing strategies, and treatment protocols have not been clearly defined.

Phytochemicals and natural compounds show multi-pathway activity in experimental models, but their clinical effectiveness is often limited by bioavailability and variability in formulation. As such, their role should currently be interpreted as adjunctive and investigational.

### 7.5 Redox-guided regenerative medicine

Oxidative stress is increasingly recognized as a barrier to successful periodontal and peri-implant regeneration. Strategies such as antioxidant preconditioning of stem cells and incorporation of redox-modulating biomaterials have demonstrated potential in experimental studies.

However, these approaches remain largely preclinical, and their clinical relevance is yet to be established. Further research is needed to determine whether modulation of the redox microenvironment can consistently enhance regenerative outcomes in humans.

### 7.6 Redesigning clinical trials in periodontology

The inconsistent clinical translation of antioxidant therapies highlights the need for improved study design. Future trials should aim to:

Incorporate standardized and validated oxidative stress biomarkers
Stratify participants based on baseline biological profiles
Evaluate long-term clinical outcomes
Control for confounding systemic and lifestyle factors

Such approaches are essential to clarify whether targeting oxidative stress provides clinically meaningful benefits beyond current standard therapies.

### 7.7 Ethical, educational, and policy implications

The integration of emerging technologies such as redox diagnostics and AI raises important ethical and practical considerations, including data reliability, standardization, and clinical interpretability. In addition, the incorporation of redox biology into dental education and clinical training will be necessary before these approaches can be responsibly implemented.

## 8 Conclusion

Periodontitis and peri-implantitis are no longer understood as purely microbial-driven diseases but as complex, multifactorial disorders arising from a dysregulated host response to a dysbiotic biofilm. Among the diverse biological processes involved, oxidative stress has emerged as a unifying pathogenic axis that bridges microbial challenge, immune dysregulation, and irreversible tissue destruction. Excessive and sustained ROS production not only inflicts direct molecular damage on lipids, proteins, and nucleic acids but also acts as a powerful amplifier of redox-sensitive inflammatory signaling pathways, perpetuating chronic inflammation and accelerating connective tissue degradation and bone resorption.

This review has synthesized current mechanistic and translational evidence demonstrating that oxidative stress is not merely a bystander phenomenon but a central disease-modifying factor in both periodontitis and peri-implantitis. The failure of endogenous antioxidant defense systems—driven by impaired enzymatic activity, dysfunctional Nrf2 signaling, mitochondrial abnormalities, and systemic modifiers—creates a pro-oxidant microenvironment that compromises tissue resilience, regenerative capacity, and therapeutic responsiveness. This redox imbalance also provides a biological basis for the strong and bidirectional links between periodontal disease and systemic conditions such as diabetes mellitus, cardiovascular disease, neuroinflammation, and aging.

Importantly, oxidative stress represents a highly actionable therapeutic target. Antioxidant-based interventions, whether systemic, locally delivered, nano-formulated, or biomaterial-incorporated, have demonstrated promising potential to attenuate inflammatory burden, protect host tissues, and enhance regenerative outcomes. When integrated with conventional mechanical and antimicrobial therapies, redox-modulating strategies may redefine periodontal and peri-implant care by shifting the focus from damage control to biological restoration and long-term disease modulation.

Beyond therapy, the emergence of redox biomarkers in saliva, gingival crevicular fluid, and peri-implant sulcular fluid offers unprecedented opportunities for early diagnosis, disease stratification, and personalized treatment planning. These biomarkers capture real-time biological activity, enabling a transition from static, damage-based assessments toward dynamic, biology-driven clinical decision-making.

Looking forward, the convergence of redox biology, nanotechnology, artificial intelligence, and regenerative medicine heralds a new era of precision periodontology and peri-implant medicine. Future research must prioritize rigorously designed, biomarker-guided clinical trials, the development of smart delivery systems, and the integration of multi-target host-modulatory strategies. Such approaches will be essential for translating molecular insights into durable clinical benefit.

In summary, oxidative stress is a central, mechanistically grounded, and clinically actionable component of periodontal and peri-implant pathogenesis. Targeting redox imbalance—rather than merely suppressing microbial load—offers a transformative framework for personalized, predictive, and preventive oral healthcare.
